# Stress and coping strategies of families of pediatric solid organ transplant recipients in times of pandemic

**DOI:** 10.3389/fpsyg.2023.1067477

**Published:** 2023-01-26

**Authors:** Mireia Forner-Puntonet, Laura Gisbert-Gustemps, Eudald Castell-Panisello, Mauricio Larrarte, Jesús Quintero, Gema Ariceta, Ferran Gran, Ignacio Iglesias-Serrano, Annabella Garcia-Morán, Gemma Español-Martín, Pol Ibañez-Jimenez, Josep Antoni Ramos-Quiroga

**Affiliations:** ^1^Department of Mental Health, Hospital Universitari Vall d’Hebron, Barcelona, Catalonia, Spain; ^2^Group of Psychiatry, Mental Health and Addictions, Vall d’Hebron Research Institute (VHIR), Barcelona, Catalonia, Spain; ^3^Department of Psychiatry and Forensic Medicine, Universitat Autònoma deBarcelona, Catalonia, Spain; ^4^Biomedical Network Research Centre on Mental Health (CIBERSAM), Madrid, Spain; ^5^Pediatric Hepatology and Liver Transplant Department, Hospital Universitari Vall d'Hebron, Universitat Autònoma de Barcelona, Barcelona, Catalonia, Spain; ^6^Pediatric Nephrology Department, Hospital Universitari Vall d’Hebron, Universitat Autònoma de Barcelona, Barcelona, Catalonia, Spain; ^7^Pediatric Cardiology Department, Hospital Universitari Vall d’Hebron, Barcelona, Catalonia, Spain; ^8^Pediatric Respiratory Medicine Department, Hospital Universitari Vall d’Hebron, Barcelona, Catalonia, Spain

**Keywords:** pediatric transplantation, solid organ transplantation, family stress, coping strategies, COVID-19, pandemic

## Abstract

**Objective:**

Pediatric solid organ transplantation (SOT) is a chronic condition that impacts the whole family system. The objective of this study is to evaluate psychopathology, family stress, and coping strategies in families of SOT recipients compared to families of healthy children and adolescents. Moreover, it analyzes if the stress related to the COVID-19 pandemic has had an additional impact on these families.

**Methods:**

The sample was recruited between May and July 2021, during the fourth and fifth wave of the pandemic in Spain. It consisted of 102 families, 51 with a pediatric recipient who had undergone a SOT (liver, kidney, heart, or lung) and 51 healthy controls, matched by child age and gender. A primary caregiver from each family answered an online sociodemographic questionnaire and different tests to evaluate family stress, depression, anxiety, coping strategies, and effects of the pandemic on the family.

**Results:**

Caregivers were mostly mothers (89.2%). Families of SOT recipients showed greater anxiety (*U* = 863.5, *p* = 0.003) and more total stress, stress related to childcare (*t* = −2.043; *p* = 0.045), and parent–child interaction stress (*U* = 355.5, *p* = 0.015). SOT families used more avoidance strategies, specifically denial (*U* = 889.5; *p* = 0.010) and abandonment of coping efforts (*U* = 1,013; *p* = 0.047), more religious strategies (*U* = 792.5; *p* = 0.031), and fewer social support coping strategies (*t* = 2.098; *p* = 0.038). No differences were found between groups in terms of exposure, impact, and distress more than 1 year after the start of the pandemic.

**Conclusion:**

SOT families showed clinical levels of anxiety, more parent–child interaction stress, more difficulties in taking care of their child, more avoidance and religious strategies, and less use of social support strategies, even 4 years after transplantation. The pandemic did not have an additional differential effect on SOT families. Caregivers of SOT patients can benefit from psychological interventions focused on parents’ mental health, parent–child connectedness, skill building, and social support aid groups, with attention to multiculturalism and promoting a better balance between caregivers. There is a need for family interventions that are maintained over time. Strategies that offer this support to families through digital resources can facilitate adjustment to chronic illness, especially in pandemic times.

## Introduction

Pediatric solid organ transplantation (SOT) is the treatment of choice for end-stage organ failure (Imam et al., 2020), which allows the medical situation of a child to change from experiencing a life-threatening disease to having a chronic health condition (Annunziato et al., 2012). Caregivers are an essential figure in SOT; even so, we still have limited understanding of the experience of caregiving through the entire process of transplantation, and there are limited data examining the long-term impact. Therefore, it is a high research priority (Jesse et al., 2021).

In general, parents of children with chronic illnesses experience greater parenting stress than parents of healthy children (Cousino and Hazen, 2013). Specifically, in pediatric SOT, although it improves quantity and quality of life, pre-transplantation and post-transplantation produce many stressors and burdens for patients and families (Cousino et al., 2017). The long-term nature of transplantation and post-transplant medical care creates more stress than what is encountered in families that go through a single surgery event, resulting in prolonged parental stress (Farley et al., 2007).

Although these families may demonstrate resilience in coping with stressors, the demanding challenges and responsibilities beyond the usual concerns of raising a child and the uncertainty regarding the transplant process may negatively impact family functioning (Cousino and Hazen, 2013). For instance, caregivers of SOT recipients show stress related to communicating, decision-making, balancing the demands of multiple roles, and facing the emotional consequences of parenting a child with an illness. Moreover, after transplantation, children can have complex emotional and physical needs, which can result in greater strain on the family system (Farley et al., 2007).

Greater parenting stress is associated with psychological distress, e.g., depressive and anxious symptomatology in the case of caregivers of children with chronic illness (Cousino and Hazen, 2013), with anxiety being the most frequent psychological disorder (Toledano-Toledano and Moral de la Rubia, 2018). In a recent study, Cushman et al. (2021) found an improvement in children’s health-related quality of life from pre-transplantation to 6 months post-transplantation, but caregivers’ psychological depression, anxiety, and global stress symptoms did not similarly improve.

Caregivers’ adaptive coping can be a protective factor in the process of children adjustment to a chronic illness (Toledano-Toledano and Moral de la Rubia, 2018). Caregivers’ optimistic and self-confident coping strategies have been associated with fewer internalizing symptoms and a better quality of life in adolescents during the process of transplantation (Yilmaz Kafali et al., 2021). However, the use of more emotion-focused coping behaviors (e.g., avoidance) in caregivers of children with chronic illness was associated with greater parenting stress (Rodenburg et al., 2007) and poorer psychosocial adaptation (Livneh, 2019).

Additionally, there is a consistent association between parent and family functioning and child health-related factors, such as adherence, barriers to medications, and number of hospitalizations (Cousino et al., 2017). These results reinforce the importance of evaluating family functioning, stress, resources, and psychopathology to offer specific treatments. Currently, in Spain, psychological support or treatment for families of pediatric transplant patients is scarce. A pre-transplant assessment and some initial post-transplant follow-up are usually carried out. However, interventions with greater continuity over time are lacking, particularly considering that it is a chronic disease that can affect children and family day-to-day life.

Finally, we must point out that the COVID-19 pandemic has been a major burden on families (Fegert et al., 2020); parents of children with physical or mental conditions in particular have reported more parental burnout and less perceived social support (Fontanesi et al., 2020). In line with this, Zhao et al. (2020) found that these caregivers presented higher prevalence of depressive and anxious symptoms in a pandemic context. In a recent study, Forner-Puntonet et al. (2021) found that, during the first wave of the pandemic, families of SOT recipients had exposed themselves less and protected themselves more from COVID-19 than the families of healthy children did, probably because they were used to prevention measures and saw contagion as a graver risk. Additionally, the families of SOT recipients perceived isolation and prevention measures as being protective and destigmatizing.

The first aim of this study is to evaluate and compare the levels of parenting stress, anxiety, and depression and coping strategies in families of SOT recipients and in healthy controls. The second aim is to analyze if the pandemic has had an additional impact on families. We hypothesize that SOT families will show higher levels of stress, depression, and anxiety. Moreover, we hypothesize that the coping strategies used to face uncertainty will be different in families of SOT pediatric recipients in comparison with the controls. Concerning the effects of COVID-19, we believe that the impact of the pandemic on these families more than 1 year after its beginning will be similar to the impact on the controls.

## Materials and methods

### Participants

The total study sample consisted of 102 caregivers, 51 of whom represented families where one member had undergone a pediatric transplant process (SOT group) and 51 families of healthy children and adolescents (control group). The inclusion criteria were: (1) for the SOT group, being an adult primary caregiver in a family of a pediatric patient, with clinical stability, who was 18 years of age or younger and had gone through a SOT (liver, kidney, heart, or lung) (2) for the control group, being an adult primary caregiver in a family with a child or adolescent 18 years or younger without chronic illness, (3) having user-level computer skills, and (4) agreeing to take part in the study. Language barriers in respondents were set as exclusion criteria, since all the questionnaires were in Spanish. The approval of the Ethics Committee (PR(AG)195/2021) of the Hospital Universitari Vall d’Hebron was obtained for the research.

### Procedure

The sample was recruited between May and July 2021, during the fourth and fifth wave of the COVID-19 pandemic in Spain. We used convenience sampling; the sample of the SOT group was obtained through families of consecutive patients who consulted the different pediatric transplant teams (liver, kidney, heart, or lung) at the Hospital Universitari Vall d’Hebron in person or on-line. Pediatric teams explained the study goals, participation rights, and data treatment procedure, and asked primary self-perceived caregivers, who could only complete the survey after giving their consent to participate. They were contacted twice by emails that included access to a web survey application, which provided the sociodemographic questionnaire and the assessment instruments. In total, 59 caregivers belonging to a family with a SOT recipient were contacted, and 51 of them responded to the full survey (86% answer rate).

To recruit the control group participants, all the SOT group families agreed to forward the received email to one or two families with a child of the same age and gender as their child. They sent it to a total of 78 caregivers of healthy children of whom 57 answered the full survey (73% answer rate). Control group participants were matched with SOT group participants by their children’s age and gender and were selected in order of response.

### Instruments

The following data were collected regarding the sociodemographic and clinical characteristics of the primary caregiver: age, gender, race/ethnicity, education level, marital status, number of children, and relationship with the child or adolescent. Regarding child or adolescent information, the following information was requested: age, gender, basic disease for which the child or adolescent had required a transplant, type of pediatric SOT, and age at transplantation, if applicable.

The Parenting Stress Index-Short Form (PSI-SF; Abidin, 1995) is a self-report tool for parents of children until 12 years old that identifies the sources and types of stress that come with parenting. It has been validated in Spanish by Díaz-Herrero et al. (2010). The PSI-SF has 36 items that use a five-point Likert-scale; it is divided into three subscales and yields a Total Stress score. The subscales are: (1) Parental Distress—the extent to which parents feel competent, restricted, conflicted, supported, and/or depressed in their roles as a parent; (2) Parent–Child Dysfunctional Interaction—the extent to which parents feel satisfied with their child and their interactions with them; and (3) Difficult Child – how parents perceive their child in terms of whether the child is easy or difficult to take care of. The Total Stress score indicates the overall level of stress a person is feeling in their role as a parent. When analyzing parental stress scores, it is important to keep in mind that some degree of parenting stress is normal. Percentiles between 15 and 80 are considered typical, high stress scores range from 81 to 84, and clinically significant levels of stress are above 85 or 90, depending on the subscale.

The Hospital Anxiety and Depression Scale (HADS; Zigmond and Snaith, 1983) is a widely used self-administered questionnaire to evaluate anxiety and depression. It has been validated in Spanish by Herrero et al. (2003). It is comprised of seven items on anxiety and seven items on depression; each item is rated on a Likert scale from 0 to 3. They are scored as being without anxiety or depression to having mild, moderate, or severe anxiety or depression.

The COPE Inventory (Carver et al., 1989) is a multidimensional coping inventory to assess the different ways in which people respond to stress. The inventory consists of 60 items to which participants respond how frequently they used each coping strategy, rated on a four-point scale (between “usually do not do this at all” to “usually do this a lot”). Crespo and Cruzado (1997) have adapted, standardized, and validated the instrument for Spanish samples. The COPE Inventory is formed by 15 subscales: Social Support, Religion, Humor, Substance use, Planification and Active Coping, Abandonment of Coping Efforts, Focus on and venting of emotions, Acceptance, Denial, Restraint, Concentrate efforts on solving the situation, Personal Growth, Positive Reinterpretation, Distraction, and Evasion.

The COVID-19 Exposure and Family Impact Survey (CEFIS) was developed by the Center for Pediatric Traumatic Stress (2020), using a rapid iterative process, in late March/early April 2020, and it has been validated by Enlow et al. (2022). The CEFIS is available in English and Spanish and should be completed by a caregiver. The CEFIS assesses exposure, impact, and distress of COVID-19 in the family. Exposure consists of 25 items with dichotomous responses (yes/no) that measures the participants’ degree of exposure to COVID-19 and related events. The Total Exposure score is the sum of all the items. Impact consists of 10 items using a four-point Likert scale (also offering a “does not apply” choice) to rate the impact on the participant’s and family’s life. Distress scale consists of two 10-point distress scales to assess how much distress caregivers and their children have experienced due to the pandemic. Item responses are averaged within each scale to yield the Impact and Distress scale scores. Higher scores indicate mean greater levels of exposure, impact, and distress. The CEFIS asks the respondents to answer by thinking about the period from March 2020 to the present, therefore addressing the full time period since the pandemic has had consequences in western countries.

### Data analysis

All data collected were analyzed using SPSS version 28.0. First, a descriptive synthesis of the main variables was performed. The analysis of the normality condition was carried out using Shapiro–Wilk statistics, the result of which guided subsequent calculations by parametric (PSI-SF: Parental Distress, Difficult Child, Total Stress; COPE: Social Support, Planification and Active Coping, Restraint, Concentrate Efforts on Solving the Situation; CEFIS: Exposure total score, Distress in Parents, and Distress scale) or non-parametric route. In the first, the comparison of two means was determined with Student’s *t*-tests and the correlational study was developed with Pearson’s test. In the non-parametric way, the comparison of two means was analyzed with Mann–Whitney *U* tests, and the correlations with Spearman’s test. Chi-squared tests were run in order to compare outcomes between groups in qualitative variables and evaluate the possibility of interaction between factors. The level of significance was set at 0.05 in all analyses. Cohen’s d was used to determine effect sizes.

## Results

Sociodemographic characteristics from caregivers and children in each group are presented in Table 1. All caregivers who responded were the children’s parents, mostly mothers (89.2%). No differences were found between groups in terms of the caregivers’ gender, age, race/ethnicity, marital status, number of children, or relationship with the child. Significant differences in the level of education (*χ*^2^ = 8.14; *p* = 0.04) were found between caregivers of both groups. Of the children, 56.9% of the children were girls and the average age of the children sample was 9.5 ± 5.3 years. The SOT group included the pediatric recipients of 22 livers, 14 kidneys, 9 hearts, and 6 lungs, with an average age at transplantation of 5.2 ± 4.9. On average, the children in the sample went through transplantation more than 4 years ago.

**Table 1:** Sociodemographic characteristics of the sample.

Variables	Total sample *n* = 102 *N* (%) or *M* ± SD	SOT group *n* = 51 *N* (%) or *M* ± SD	Control group *n* = 51 *N* (%) or *M* ± SD	*χ*^2^ or *t*	Value of *p*
Caregiver gender (relationship with the child)				0.10	0.75
Male (father)	11 (10.8)	5 (45.5)	6 (54.5)		
Female (mother)	91 (89.2)	46 (50.5)	45 (49.5)		
Caregiver age	43.44 ± 15.97	43 ± 6.12	44 ± 5.84	0.64	0.523
Caregiver race					0.061
Caucasian	92 (90.2%)	43 (84.3%)	49 (96.1%)		
African American	5 (4.9%)	5 (9.8%)	0		
Hispanic	5 (4.9%)	3 (5.9%)	2 (3.9%)		
Caregiver education				8.14	0.04
Primary to High School	29 (28.4)	21 (72.4)	8 (27.6)		
University and Postgraduate	73 (71.6)	30 (41.1)	43 (58.9)		
Marital status				0.10	0.95
Single	10 (9.8)	5 (50)	5 (50)		
Married/Cohabiting	81 (79.4)	40 (49.4)	41 (50.6)		
Separated/Divorced	11 (10.8)	6 (54.5)	5 (45.5)		
Number of children	1.8 ± 0.7	1.7 ± 0.8	1.8 ± 0.6	0.70	0.484
Age of pairing children	9.5 ± 5.3	9.5 ± 5.3	9.5 ± 5.4	−0.07	0.945
Gender of pairing children				0	1
Male	44 (43.1)	22 (50)	22 (50)		
Female	58 (56.9)	29 (50)	29 (50)		
Transplant type				–	–
Liver	22 (43.1)	22 (43.1)	0		
Kidney	14 (27.5)	14 (27.5)	0		
Heart	9 (17.6)	9 (17.6)	0		
Lung	6 (11.8)	6 (11.8)	0		
Age at the time of transplantation	5.2 ± 4.9	5.2 ± 4.9		–	–

*M*, Mean; SD, Standard Deviation; SOT group, Solid Organ Transplant group; *χ*^2^, Chi-squared test; *t*, Student’s *t*-test.

### Parenting stress

Although all scores were in a non-clinical range, between percentiles 15 and 80, the families in the SOT group showed greater Total Stress than the control group (*t* = −2.083; *p* = 0.041) and significantly higher scores on the Parent–Child Dysfunctional Interaction (*U* = 355.5; *p* = 0.015) and Difficult Child scales (*t* = −2.043; *p* = 0.045), with moderate effect sizes (see Table 2). These results were maintained after controlling for the level of education. However, there were no significant differences on Parental Distress between the groups.

**Table 2:** Parenting Stress Index-Short Form (PSI-SF).

Variables	Total sample *n* = 66 *M* ± SD	SOT group *n* = 33 *M* ± SD	Control group *n* = 33 *M* ± SD	*t* or *U*	Value of *p*	Cohen’s *d*
Parental Distress (PD)	29.9 ± 9.7	29.2 ± 9.9	30.6 ± 9.6	0.56	0.578	0.144
Parent–Child Dysfunctional Interaction (P-CDI)	21.4 ± 6.6	23.4 ± 6.7	19.4 ± 5.8	355.50	0.015	−0.638
Difficult Child (DC)	27.3 ± 8.5	29.4 ± 8.8	25.2 ± 7.8	−2.04	0.045	−0.505
Total stress	78.1 ± 15.5	82 ± 16.5	74.2 ± 13.7	−2.08	0.041	−0.514

Included families of children until 12 years old. *M*, Mean; SD, Standard Deviation; SOT group, Solid Organ Transplant group; *t* = Student’s *t*-test; *U* = Mann–Whitney *U*-test.

### Anxiety and depression of caregivers

Table 3 summarizes the quantitative data extracted from the HADS related to the anxiety and depression of caregivers. Regarding anxiety, the SOT group reported significantly higher levels than the control group (*U* = 863.5; *p* = 0.003), with moderate effect sizes, showing anxious symptomatology on probable case levels. Families in the SOT group reported themselves to be more tense; feeling sort of frightened, as if something awful was about to happen; feeling more worried, finding it difficult to relax; and experiencing sudden feelings of panic. These results were maintained after controlling for the level of education. No significant differences were found between the groups on the Depression Scale.

**Table 3:** Hospital Anxiety and Depression Scale (HADS).

Variables	Total sample *n* = 102 *M* ± SD	SOT group *n* = 51 *M* ± SD	Control group *n* = 51 *M* ± SD	*U*	Value of *p*	Cohen’s *d*
Anxiety scale	6.6 ± 4.2	7.9 ± 4.3	5.4 ± 3.8	863.50	0.003	−0.616
Depression scale	4.2 ± 3.7	4.9 ± 4	3.5 ± 3.3	1032.50	0.071	−0.382

*M*, Mean; SD, Standard Deviation; SOT group, Solid Organ Transplant group; *U* = Mann–Whitney *U*-test.

### Coping strategies

Table 4 summarizes the results of the COPE Inventory. SOT families used significantly more denial strategies (*U* = 889.5; *p* = 0.010), used religion as a coping strategy more (*U* = 792.5; *p* = 0.031), and tended to abandon coping efforts (*U* = 1,013; *p* = 0.047), with small effect sizes. Families in the control group used more social support as a coping strategy (*t* = 2.098; *p* = 0.038) with small effect sizes. Nevertheless, all responses were in a normative clinical range. These results were maintained after controlling for the level of education.

**Table 4:** COPE inventory.

Variables	Total sample *n* = 102 *M* ± SD	SOT group *n* = 51 *M* ± SD	Control group *n* = 51 *M* ± SD	*t* or *U*	Value of *p*	Cohen’s *d*
Social support	21.9 ± 5.8	20.8 ± 5.3	23.1 ± 6.0	2.10	0.038	0.406
Religion	5.6 ± 2.0	5.9 ± 1.9	5.2 ± 2.1	792.50	0.031	−0.35
Humor	7.4 ± 2.4	7.0 ± 2.3	7.8 ± 2.3	1061.50	0.107	0.348
Substance use	4.2 ± 0.6	4 ± 0	4 ± 1	1285.50	0.867	0
Planification and active coping	16.4 ± 2.8	16.0 ± 2.4	16.7 ± 3.1	1.22	0.226	0.253
Abandonment of coping efforts	4.7 ± 1.2	4.9 ± 1.3	4.4 ± 1.1	1013.00	0.047	−0.415
Focus on and venting of emotions	8.4 ± 2.3	8.2 ± 2.1	8.7 ± 2.4	1125.00	0.386	0.222
Acceptance	11.8 ± 2.2	11.7 ± 2.4	11.9 ± 2.1	1248.50	0.724	0.089
Denial	5.6 ± 1.8	6.1 ± 2.0	5.2 ± 1.6	889.50	0.010	−0.497
Restraint	9.7 ± 2.1	9.5 ± 1.9	10.0.0 ± 2.3	1.26	0.209	0.237
Concentrate efforts on solving the situation	10 ± 2.4	9.8 ± 2.3	10.2 ± 2.4	0.67	0.504	0.17
Personal growth	6.5 ± 1.1	6.6 ± 1.0	6.5 ± 1.1	1121.00	0.564	−0.095
Positive reinterpretation	8.9 ± 1.6	8.8 ± 1.6	9.1 ± 1.6	1105.00	0.232	0.187
Distraction	6.6 ± 1.0	6.4 ± 0.9	6.7 ± 1.0	697.50	0.125	0.21
Evasion	5.3 ± 1.4	5.4 ± 1.5	5.2 ± 1.3	1195.00	0.47	−0.142

*M*, Mean, SD, Standard Deviation; SOT group, Solid Organ Transplant group; *t*, Student’s *t*-test; *U* = Mann–Whitney *U*-test.

### Exposure, impact, and distress of COVID-19

More than 1 year after the beginning of the pandemic, no significant differences were found between both groups in terms of exposure, impact, or distress related to the pandemic (see Table 5).

**Table 5:** COVID-19 exposure and family impact survey (CEFIS).

Variables	Total sample *n* = 102 *M* ± SD	SOT group *n* = 51 *M* ± SD	Control group *n* = 51 *M* ± SD	*t* or *U*	Value of *p*	Cohen’s *d*
Exposure total score	8.8 ± 2.7	8.7 ± 2.9	9.0 ± 2.5	0.660	0.511	0.111
Impact total score	1.9 ± 1.3	1.9 ± 1.3	1.8 ± 1.3	1250.50	0.735	−0.077
Distress in parents	5.6 ± 2.3	5.9 ± 2.4	5.4 ± 2.2	−1.038	0.302	−0.217
Distress in children	4.8 ± 2.6	4.7 ± 2.6	5.0 ± 2.5	1207.00	0.528	0.118
Distress scale	5.2 ± 2.1	5.0 ± 2	5.3 ± 2.2	−0.235	0.815	0.143

*M*, Mean; SD, Standard Deviation; SOT group, Solid Organ Transplant group; *t*, Student’s *t*-test; *U* = Mann–Whitney *U*-test.

## Discussion

To our knowledge, this is the first study to analyze family stress and coping strategies of SOT families in times of pandemic. In addition, although SOT is a life-threatening event that not only impacts the patients themselves but their entire family systems, there are very few studies that delve into family stress, and we did not identify any study that focuses on the coping strategies of caregivers of children and adolescents who have undergone transplantation.

First of all, regarding the sociodemographic variables, it is important to remark that the majority of respondents and primary caregivers in our study were mothers. These results are in line with those of Cardinali et al. (2019), which pointed out that mothers usually assume the role of primary caregivers, implying a greater interference with and more limitations in their work and social life, as well as in other life domains. It is important to explore specific gender differences regarding caregiving for a child who has undergone SOT in order to reduce gender stereotypes that are still present in parents’ perceptions and to promote a better balance in caregiving (De Piccoli et al., 2016). Therefore, a collaborative relationship between all the main caregivers and SOT pediatric recipients is essential, as it is a space to share information and knowledge about procedures and illness, offer support on emotional regulation, or develop skills to help the child and the whole family meet existing challenges.

In relation to the first aim of the study, families of pediatric transplantation recipients reported normal levels of parenting stress, although they showed higher scores on the Difficult Child scale, the Parent–Child Dysfunctional Interaction Stress scale, and the Total Stress score than the controls did. Higher scores on the Difficult Child scale are related to more stress in taking care of their child and to possible difficulties in managing their child’s behavior. Higher scores on the Parent–Child Dysfunctional Interaction scale indicate that it is more difficult for those parents to create a proper bond with their child. These difficulties are probably related to child and parent anxiety, the physical effects of the illness, and the treatment conditions for chronic illness (Ødegård, 2005). According to Golfenshtein et al. (2016), interventions that focus on the improvement of the parent–child relationship to enhance connectedness and skill-building interventions (education, problem solving, and parental techniques) had a positive impact on parenting stress, although they have failed until now to demonstrate long-term effects thus far.

In our study, caregivers of SOT recipients showed greater levels of anxiety; these results are on a clinical borderline level, reinforcing the importance of treating these symptoms in parents in order to reduce the risk of greater impairment. Interventions for parents of children with chronic illness with a focus on the parents’ mental health have been lacking (Eccleston et al., 2015), although recent studies based on cognitive behavioral therapy and acceptance and commitment therapy showed promising results (Douma et al., 2021).

Caregivers of children and adolescents who have undergone transplantation tend to significantly drop coping efforts and use more denial strategies than the controls did, although the strategies used by the families of SOT recipients do not reach clinical levels of significance. These avoidance strategies have been described as maladaptive; they are able to promote some relief in the short term, but induce lower well-being (Waugh et al., 2021) and greater distress in the medium to long term (Cousino and Hazen, 2013). Nap-van der Vlist et al. (2021) reinforced the importance of open communication about stress as a first step to cope with these ineffective strategies and to provide parent–child positive dyadic coping (Nap-van der Vlist et al., 2021). We hypothesize that families of SOT recipients’ have learned other strategies to cope with the uncertainty and stress related to transplantation, and that some kind of avoidance behavior, in a non-clinical way, allows them to cope with some difficulties of the transplantation process, permitting them at the same time to escape a little bit from certain worries in their daily life.

However, SOT caregivers also used more religious coping strategies, which probably brought them hope and courage to cope with the process of transplantation. This kind of coping can be active (e.g., benevolent religious reappraisals) or passive (e.g., waiting for something to change or a miracle), showing that religious coping can be adaptive or maladaptive depending on its positive or negative consequences (Peres and Lucchetti, 2010). Attention to multicultural issues is an important element for families, especially bearing in mind that active behaviors linked to religion or spirituality can contribute to the adaptation to chronic illness (Pargament, 2001).

In the case of coping strategies, it is additionally important to underline that SOT families use less social support coping strategies, although social support represents a key variable associated with optimal adjustment to chronic illness (Helgeson and Zajdel, 2017) and can be a buffer to parental stress (Pinquart, 2018). We believe that the lower use of social support as a strategy by the caregivers of SOT recipients might be due to feelings or perceptions of not being completely understood or emotionally supported by people who are not going through the same process. Promoting support and accompaniment by a close family member or by social networks could be a positive factor, but health institutions in particular can mobilize additional social support – for instance, promoting mutual aid groups for SOT families to come together. Likewise, strategies to educate, inform, and offer these supports among SOT families through digital resources can facilitate adjustment to chronic illness (DeHoff et al., 2016), especially in pandemic times.

In line with our results, the Committee on Hospital Care and Institute for Patient- and Family-Centered Care (2012) from the American Academy of Pediatrics has repeatedly emphasized the need to adopt a patient- and a family-centered approach to children’s chronic conditions. In the field of pediatric transplantation, there is a lack of studies that explore the supportive needs of SOT recipients’ families; this is crucial for effective child and adolescent coping and adaptation to illness. Caregivers’ levels of anxiety, stress, and coping strategies can be screened routinely in the transplantation process. These aspects are modifiable intervention targets and can facilitate parents and children’s adjustment to illness. For instance, cognitive behavioral therapy and acceptance commitment therapy could be implemented with the caregivers of SOT recipients. Intervention programs directed to reduce anxiety to non-clinical levels, help caregivers manage the stress related to the difficulty of adjusting parent–child interaction, and promote effective, optimistic, and self-confident coping strategies could be enhanced.

Regarding the second aim of the study, more than 1 year after the beginning of the pandemic, there were no differences between the exposure, impact, and distress from the pandemic on families of pediatric SOT and controls. Therefore, the pandemic did not have an additional differential effect on SOT families after more than 1 year; the differences found in stress, anxiety, or coping strategies were probably more related to the process of transplantation. However, we cannot forget that the pandemic has had an economic impact on families and that subsidies from the government and other institutions can help to provide economic stability and security to caregivers, thus reducing anxiety and improving adjustment in the uncertain situation of transplant processes.

While the current study is an important first step in understanding caregivers’ psychopathology, family stress, and coping strategies for pediatric SOT in times of pandemic, there were some limitations. The sample size was limited, although studies with SOT recipients tend to have similar samples due to the specificity of the studied population (Cousino et al., 2017); nevertheless, the response rate was good, probably due to the close relationship between families and the medical teams and the proximity of the control families to SOT recipients. Regarding recruitment, although we believe that the acquaintance control method was useful in the pandemic situation, this method of recruitment could have influenced the homogeneity of some variables in the sample (e.g., gender). On the other hand, the control group had higher levels of education, which were controlled for statistical analysis. These differences could be due to the fact that our center is a reference in transplantation, attending to families with different levels of education from different countries, where such procedures are not carried out. Additionally, it is important to include families with diverse cultural backgrounds in future research in order to study differences in stress and coping strategies across cultures. Other family characteristics, such as whether they are single-parent families or how the burden of the childcare is distributed among primary caregivers, should also be included in future studies. Moreover, the study did not include additional validated measures to evaluate posttraumatic stress disorder on caregivers, quality of life, or resilience, which could provide a broader and more constructive picture of the topic. In terms of future lines of research, it is important to conduct follow-up studies to evaluate the levels of anxiety, stress, and coping styles of families during the entirety of the transplantation process, as well as to compare them to other chronic medical conditions, and to identify specific risk times where major stress, anxiety, and coping should be addressed. Additionally, psychological intervention studies to improve the stress level of SOT families in the follow-up are needed.

In conclusion, families of SOT patients showed more difficulties regarding parent–child interaction, more stress in taking care of their child, more avoidance coping strategies, less use of social support strategies, and clinically significant levels of anxiety, even 4 years after transplantation. However, although they initially had a different way of adapting to the pandemic (Forner-Puntonet et al., 2021), COVID-19 does not seem to add additional stress to families of SOT recipients at the current point. Caregivers of SOT patients can benefit from psychological interventions that focus mainly on parents’ mental health, parent–child connectedness, skill building, and social support aid groups, with attention to multiculturalism and promoting a better balance between caregivers, reflecting the need for family interventions that are maintained over time. Moreover, there is a critical need to evaluate the efficacy of this interventions to support the health and well-being of parents and families (Law et al., 2019) during the whole SOT process. Additionally, strategies that offer this support to families through digital resources can facilitate adjustment to chronic illness, especially in pandemic times.

## Data availability statement

The raw data supporting the conclusions of this article will be made available by the authors, without undue reservation.

## Ethics statement

The studies involving human participants were reviewed and approved by the Ethics Committee (PR(AG)195/2021) of the Hospital Universitari Vall d’Hebron was obtained for the research. Written informed consent to participate in this study was provided by the participants’ legal guardian/next of kin.

## Author contributions

All the authors have contributed to the intervention, design, acquisition, analysis, interpretation, and/or review of the work and agree to be accountable for all aspects of the work, ensuring that questions related to the accuracy or integrity of any part of the work are appropriately investigated and resolved. All authors contributed to the article and approved the submitted version.

## Conflict of interest

GA reported lectures and advisory honoraria from Chiesi, Recordati Rare Diseases, Alexion (Astra Zeneca Rare Diseases), Advicenne, Dicerna, Alnylam, and Kyowa Kirin.

GE-M has received travel grants from Takeda, Laboratorios Rubió, Lundbeck, and Angelini Pharma España for participating in psychiatric meetings.

JAR-Q was on the speakers’ bureau and/or acted as consultant for Eli-Lilly, Janssen-Cilag, Novartis, Shire, Takeda, Bial, Shionogui, Lundbeck, Almirall, Braingaze, Sincrolab, Medice, and Rubió, Raffo in the last 5 years. He also received travel awards (air tickets + hotel) for taking part in psychiatric meetings from Janssen-Cilag, Rubió, Shire, Takeda, Shionogui, Bial, Medice, and Eli-Lilly. The Department of Psychiatry chaired by him received unrestricted educational and research support from the following companies in the last 5 years: Eli-Lilly, Lundbeck, Janssen-Cilag, Actelion, Shire, Ferrer, Oryzon, Roche, Psious, and Rubió.

The remaining authors declare that the research was conducted in the absence of any commercial or financial relationships that could be construed as a potential conflict of interest.

## Publisher’s note

All claims expressed in this article are solely those of the authors and do not necessarily represent those of their affiliated organizations, or those of the publisher, the editors and the reviewers. Any product that may be evaluated in this article, or claim that may be made by its manufacturer, is not guaranteed or endorsed by the publisher.
